# Crystal structure and Hirshfeld surface analysis of 6-[4-(1-cyclo­hexyl-1*H*-tetra­zol-5-yl)but­oxy]-8-nitro-3,4-di­hydro­quinolin-2(1*H*)-one

**DOI:** 10.1107/S2056989025001793

**Published:** 2025-03-04

**Authors:** Arnab Dutta, Yogesh Dhasmana, T. P. Mohan, B. Selvakumar, Deepak Chopra

**Affiliations:** a Department of Chemistry Indian Institute of Science Education and Research Bhopal, Bhauri Bhopal 462066 India; b Bioneeds India Private Limited, P-3, Peenya Industrial Area, 1st Main Road, Peenya 1st stage, Bangalore 560094, Karnataka., India; University of Neuchâtel, Switzerland

**Keywords:** crystal structure, inter­molecular inter­action, crystal packing, mol­ecular conformation, Hirshfeld surface

## Abstract

The crystal structure of 6-[4-(1-cyclo­hexyl-1*H*-tetra­zol-5-yl)but­oxy]-8-nitro-3,4-di­hydro­quinolin-2(1*H*)-one, C_20_H_26_N_6_O_4_ (**I**), was characterized by single-crystal X-ray diffraction. The primary focus was to establish the position of the nitro group, the mol­ecular conformation, and the role of inter­molecular inter­actions towards the crystal packing of **I**. The crystalline structure is mainly consolidated by π–π, C—H⋯O, C—H⋯N, N⋯C(π) and O⋯C(π) inter­actions. The contributions of different inter­actions towards the crystal packing were further analyzed using Hirshfeld surface and fingerprint plots.

## Abstract 2

The composition of the nitro-substituted compound 6-[4-(1-cyclo­hexyl-1*H*-tetra­zol-5-yl)but­oxy]-8-nitro-3,4-di­hydro­quinolin-2(1*H*)-one has been unequivocally established from single-crystal X-ray diffraction. The mol­ecular conformation and the role of the different inter­molecular inter­actions in the crystal packing has also been explored.

## 1. Introduction

Cilostazol is an important active pharmaceutical ingredient used to treat inter­mittent claudication associated with peripheral vascular disease (Lauters & Wilkin, 2002 ▸). However, cilostazol has the potential to generate nitroso impurities. The pharmaceutical industry is subject to stringent regulations to control genotoxic nitro­samine impurities in medications, as these compounds pose significant health risks (Vikram *et al.*, 2024 ▸). Therefore, investigating the presence of nitro­samine impurities is crucial.

Given the possibility of nitroso impurity formation, it is essential to identify the specific site on cilostazol where nitro­sation may occur. In this study, we attempted to synthesize *N*-nitroso cilostazol using a standard method (Lopez-Rodriguez *et al.*, 2020 ▸). Unexpectedly, instead of obtaining the anti­cipated product – 6-[4-(1-cyclo­hexyl-1*H*-tetra­zol-5-yl)but­oxy]-1-nitroso-3,4-di­hydro­quinolin-2(1*H*)-one (*N*-nitroso cilostazol) – we isolated a different compound, referred to as **I**. This compound was fully characterized using ^1^H NMR and LC-MS, spectroscopy and single-crystal X-ray diffraction.
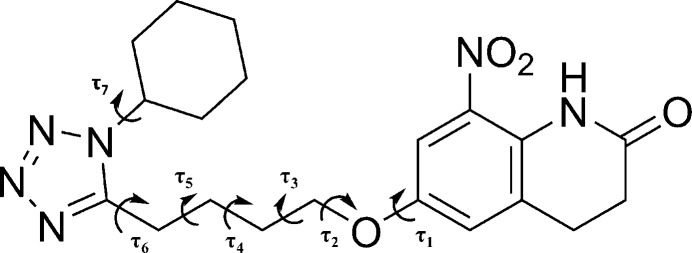


## 2. Structural commentary

**Figure 1:**
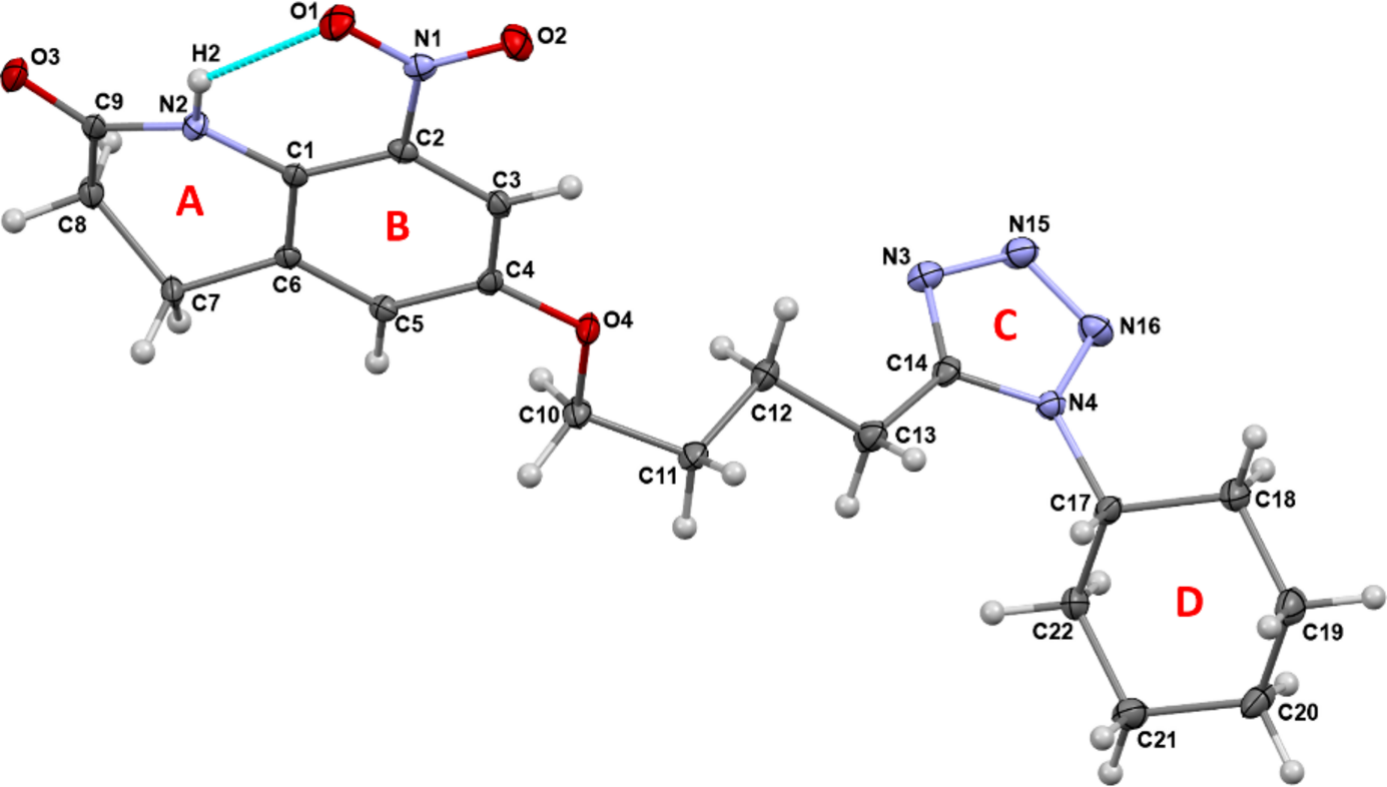
The mol­ecular structure of **I** with 50% probability level ellipsoids. The dotted line indicates the intra­molecular N2—H2⋯O1 inter­action.

The mol­ecular structure features a di­hydro­quinolinone moiety (N2/C1–C9/O3) and a tetra­zole moiety composed of two fused six-membered rings (C1–C6, **B**, and C1/C6–C9/N2, **A**) and a five-membered ring (C14/N3/N15/N16/N4, *C*), respectively (Fig. 1 ▸). The dihedral angle between the plane through ring **B** and atoms O4, C10, C11 and the mean plane through ring **C** and atoms C13, C12, C11 is 82.46 (6)°. The but­oxy chain exhibits rotational freedom, contributing to the conformational flexibility of the mol­ecule. The various torsions of the but­oxy chain are represented by τ_2_, τ_3_, τ_4_, τ_5_, and τ_6_, and listed in Table 1 ▸. The C2 and C4 carbon atoms are connected to the nitro and alk­oxy substituents, respectively, and the presence of two *sp*^3^-hybridized carbon atoms (C7 and C8) makes ring **A** non-planar. The puckering parameters (Cremer & Pople, 1975 ▸) generated by *PLATON* (Spek, 2020 ▸) were obtained for the different rings. For ring **A**, the puckering parameters are *Q* = 0.420 (2) Å, θ = 65.9 (3)° and *φ* = 25.1 (3)°, the value of θ indicating a screw-boat conformation (Boeyens, 1978 ▸). Atom N4 of ring **C** is linked to the cyclo­hexyl ring (C17–C22, *D*) [*Q* = 0.582 (3) Å, θ = 1.1 (3)° and *φ* = 138 (11)°]. The value of θ is very close to zero, thus indicating a chair conformation for ring **D**. The most acidic hydrogen atom (H2) is involved in the formation of an intra­molecular hydrogen bond with the O1 atom of the nitro group [N2⋯O1 = 2.650 (3) Å, ∠N—H⋯O = 128°].

## 3. Supra­molecular features

**Figure 2:**
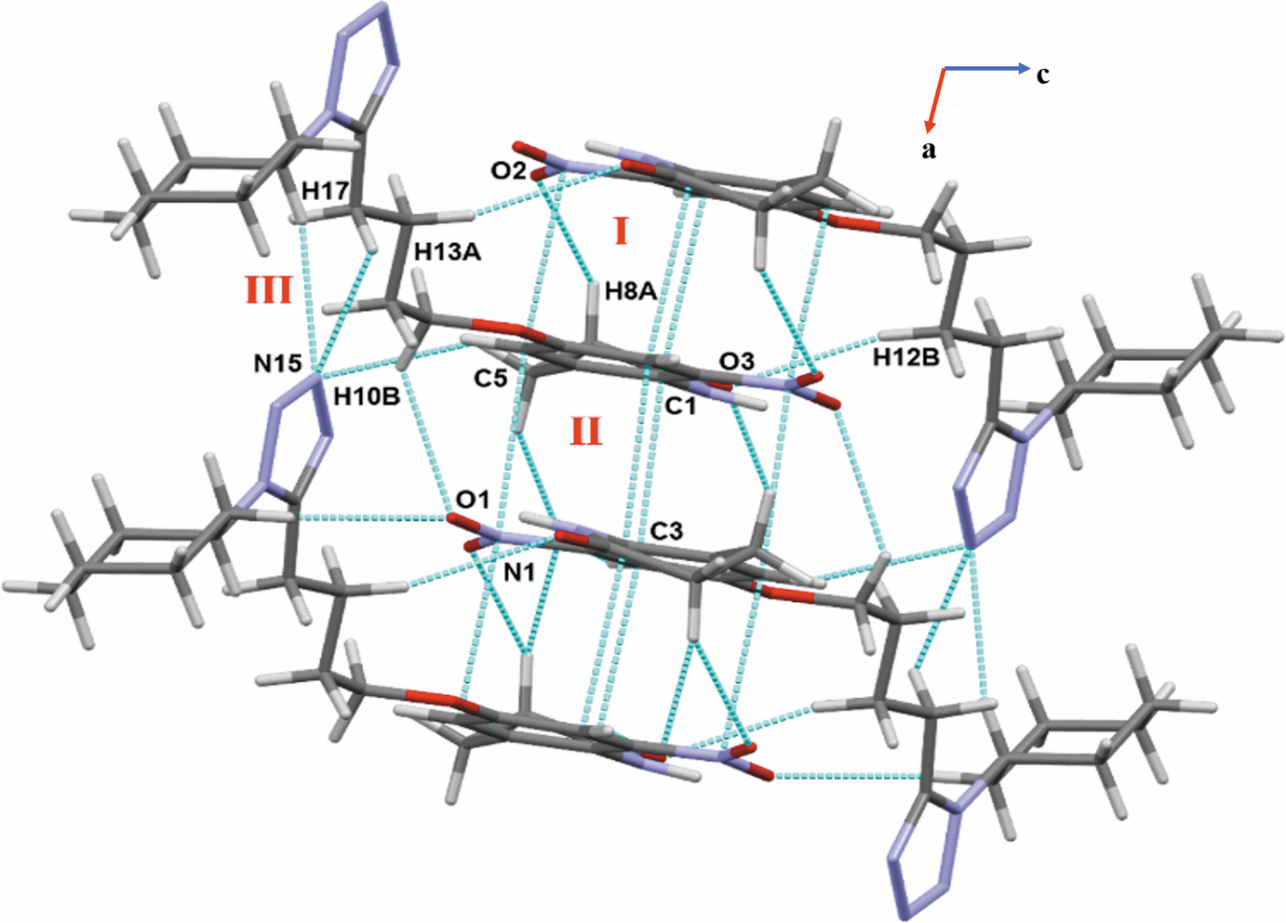
Crystal packing extending along the *a*-axis *via* π–π stacking, N(π-hole)⋯C(π), C—H⋯O and C—H⋯N inter­actions.

The crystal packing was further analysed by *Mercury* (Version 2024.1.0; Macrae *et al.*, 2020 ▸). The crystal packing along the *a*-axis shows that the mol­ecules form three motifs by inversion symmetry, I, II and III, respectively (Fig. 2 ▸ ▸, Table 2 ▸). The motifs I and II are consolidated by π–π stacking, N(π-hole)⋯C(π) and C—H⋯O hydrogen-bonding inter­actions, while motif III contains onlyC—H⋯N hydrogen bonds (involving H17 and N15 and H13*A* with N15). The π–π stacking occurs in a parallel offset fashion between **B** rings in both motifs I and II, the latter one consisting of a very short π–π inter­action between C3(π)⋯C1(π) with a distance of 3.2067 (3) Å. In the MESP map (Fig. 4 ▸*a*), the electropositive blue region over N1 (0.0332 a.u.) shows the π hole for the N1⋯C5 inter­action. In motif III, ring *C* forms C—H⋯N inter­actions with the but­oxy chain and cyclo­hexyl ring **D**, which propagate along the *a*-axis direction.

**Figure 3:**
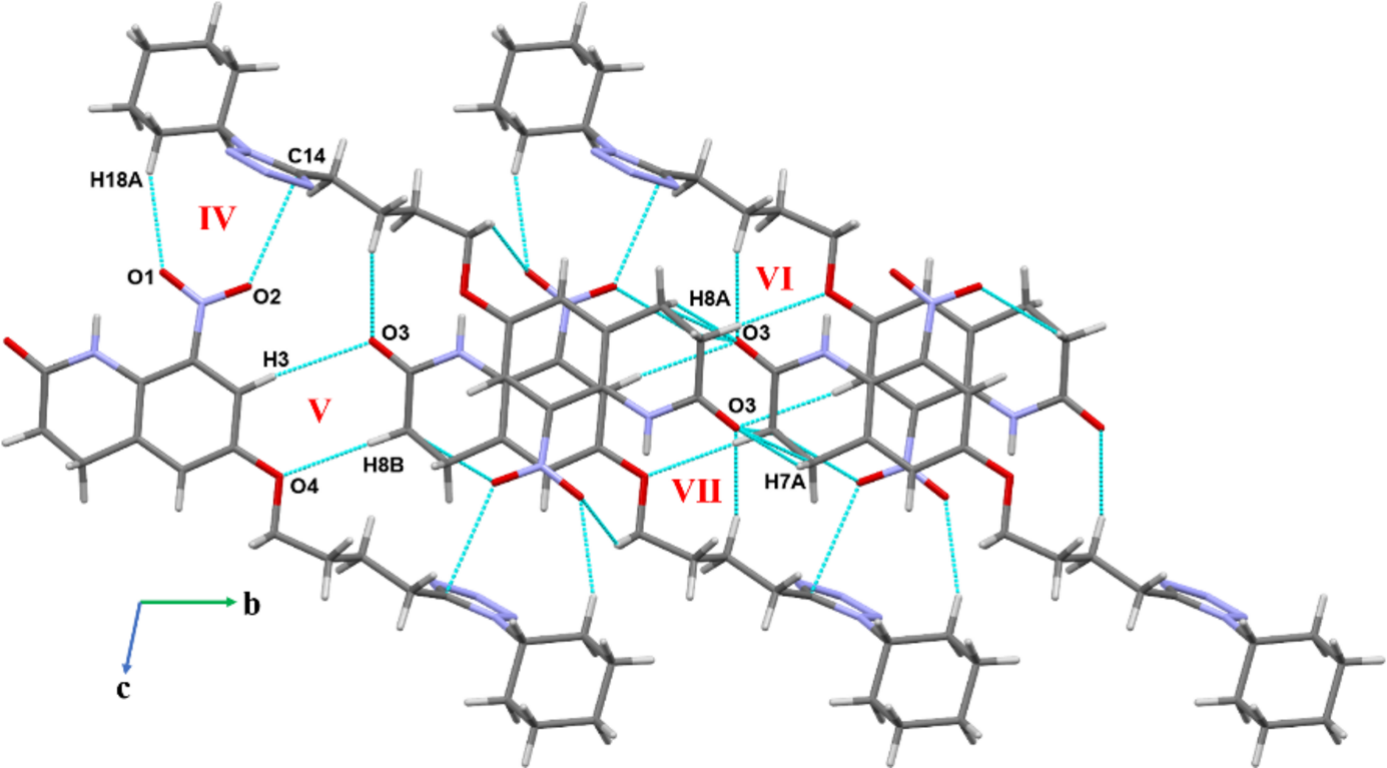
Crystal packing of **I** along the *bc* plane showing C—H⋯O and O⋯C(π) inter­actions.

Along the *bc* plane (Fig. 3 ▸), motif IV forms a centrosymmetric dimer through C—H⋯O and O⋯C(π) inter­actions, while motif V consists of C3—H3⋯O3 and C8—H8*B*⋯O4 inter­actions. Motif V utilizes translation symmetry along the *b*-axis direction for the packing of mol­ecules in the crystal. The remaining motifs VI and VII are also involved in the formation of C—H⋯O hydrogen bonds, the latter one shows a significant short contact [C7⋯O3 = 3.170 (2) Å]. It is noteworthy that atoms H3 and H17, which are attached to hybridized *sp*^2^ carbons, form the most directional C—H⋯O and C—H⋯N hydrogen bonds in the crystal packing.

## 4. Hirshfeld surface analysis and fingerprint plots

**Figure 4:**
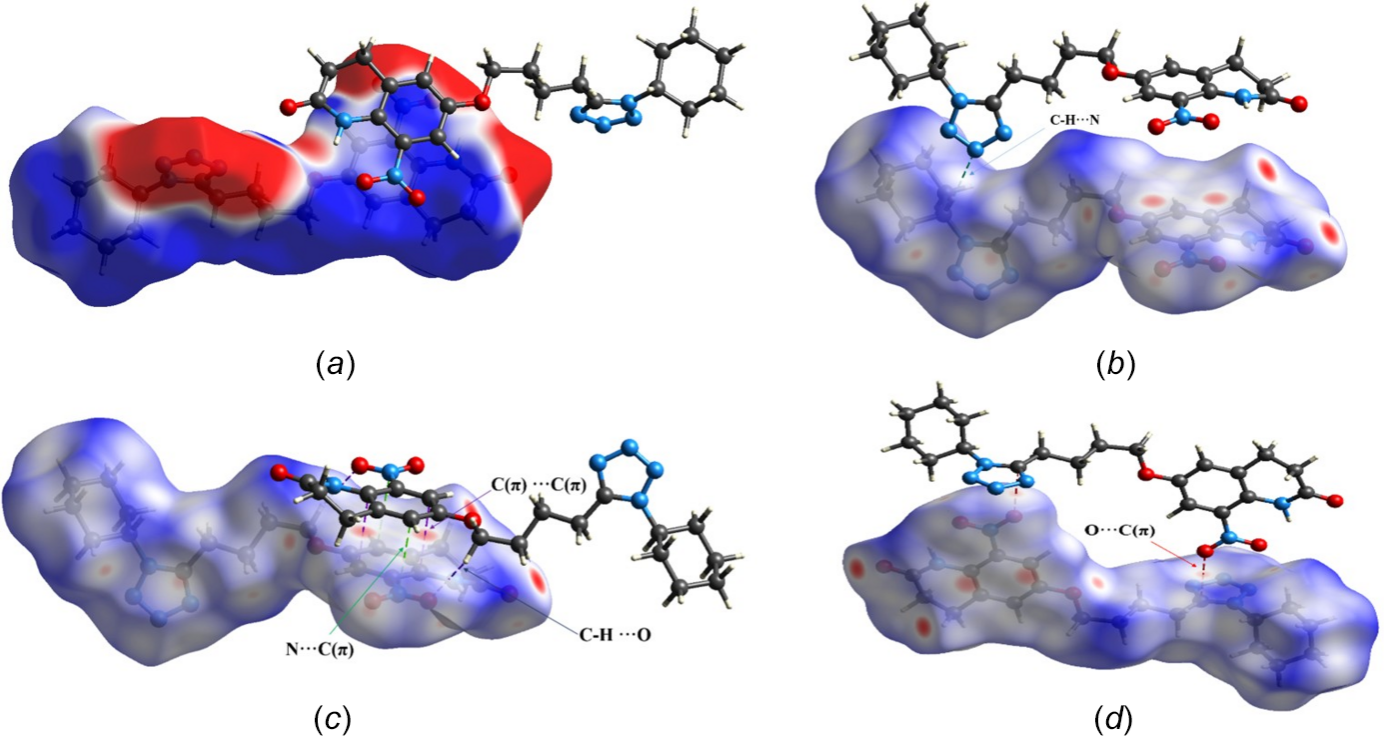
The mol­ecular electrostatic potential map (*a*) and Hirshfeld surface mapped over *d*_norm_ (*b*, *c*, *d*). Non-covalent inter­actions are indicated by dashed lines.

Hirshfeld surface analysis was performed to investigate and visualize the inter­molecular inter­actions present between mol­ecules and most importantly to qu­antify the individual contributions of different inter­actions involved in the crystal packing (Spackman *et al.*, 2021 ▸). The Hirshfeld surface and the 2D fingerprint plots (Spackman & McKinnon, 2002 ▸) were generated using *CrystalExplorer* (version: 21.5) over electrostatic potential range −0.02 a.u. to +0.02 a.u., as depicted in Figs. 4 ▸ and 5 ▸, respectively. The red spots on the Hirshfeld surface (plotted over *d*_norm_) (Fig. 4 ▸*b*,*c*,*d*) indicate the presence of inter­molecular π–π stacking, C—H⋯N, C—H⋯O, O⋯C(π) and N⋯C(π) short contacts. In the crystal packing, the relative contributions of the inter­actions are: O⋯H/H⋯O (22.2%), N⋯H/H⋯N (17.1%), C(π)⋯C(π) (3.7%), O⋯C(π)/C(π)⋯O (2.6%) and N⋯C(π)/C(π)⋯N (2.3%).

## 5. Database survey

A CSD (Groom *et al.*, 2016 ▸) survey for the cilostazol mol­ecule was performed using CCDC ConQuest (version 2024.1.0). The five resulting hits with refcodes OCIKIX, OCIKOD, OCIKUJ (Yoshimura *et al.*, 2017 ▸) and XOSGUH, XOSGUH01 (Whittall *et al.*, 2002 ▸) report co-crystals of cilostazol and the structures of polymorphic forms of cilostazol.

OCIKIX, OCIKOD and OCIKUJ (Yoshimura *et al.*, 2017 ▸) are three co-crystals of cilostazol. Cilostazol is a poorly soluble compound, and in order to increase the solubility, co-crystals of cilostazol with 4-hy­droxy­benzoic acid, 2,4-di­hydroxy­benzoic acid and 2,5-di­hydroxy­benzoic acid (1:1 stoichiometric ratio) have been prepared for different pharmaceutical applications.

**Figure 6:**
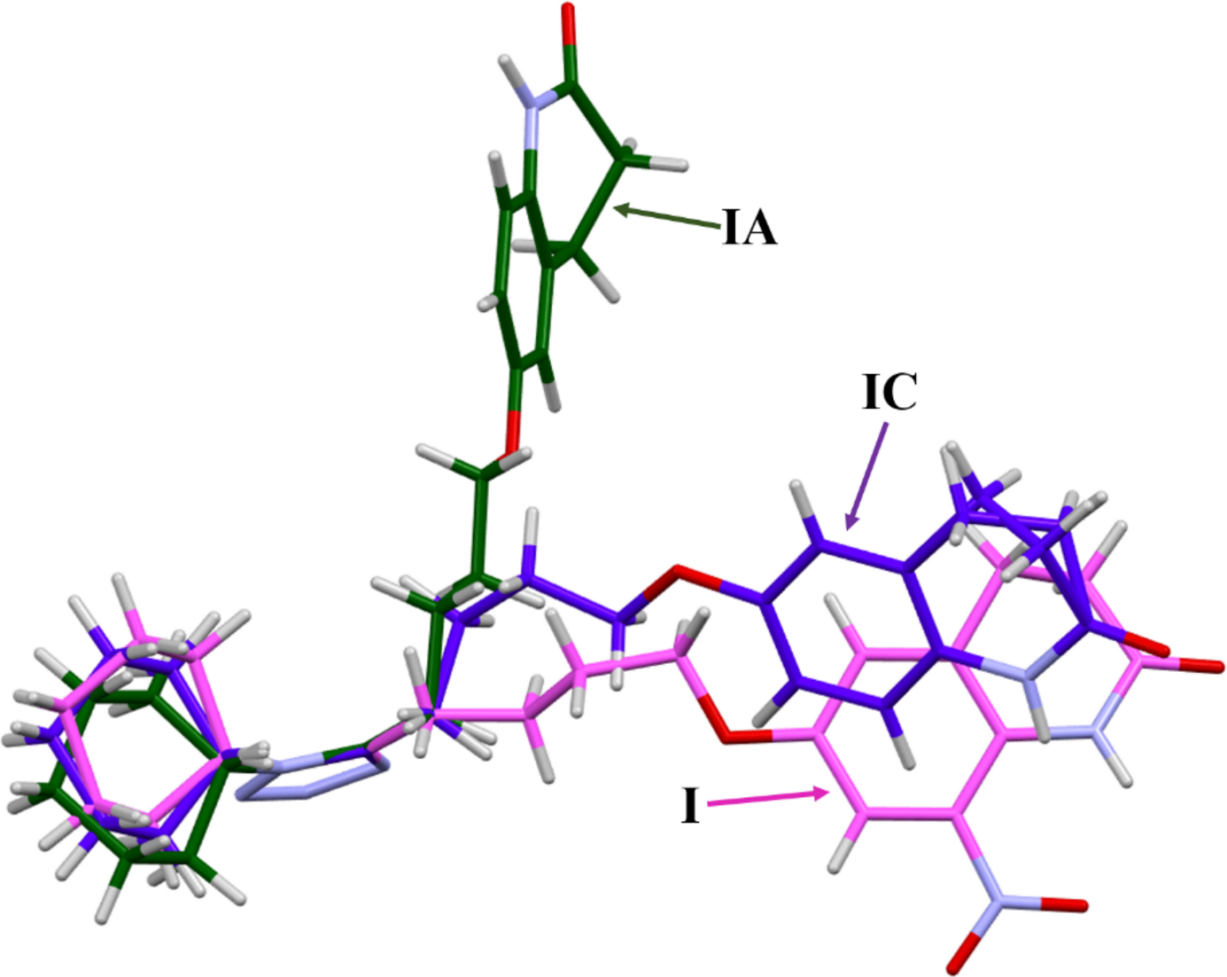
An overlay of structures **I**, **IA** and **IC**.

XOSGUH and XOSGUH01 (Whittall *et al.*, 2002 ▸) feature two unique conformational polymorphic forms of cilostazol. The study shows how the conformational differences can possibly influence the inter­molecular forces and packing of the mol­ecules during crystallization. As a result of the conformational flexibility of the but­oxy chain, cilostazol crystallizes in two different space groups, *P*2_1_/*n* (**IC**) and *Pbca* (**IA**). It is observed that **I** exists in two different conformations for the two reported polymorphs of cilostazol. Conformational variations were analysed by generating a mol­ecular overlay diagram, keeping the tetra­zole rings fixed for the three mol­ecules (Fig. 6 ▸). It is found that **I** and **IC** have similar conformations but differ significantly from **IA**. The magnitudes of the torsion angles (Table 1 ▸) indicate that the most significant conformational differences are observed in the but­oxy chains.

## 6. Synthesis and crystallization

Cilostazol (1.00 g, 2.7 mmol) was stirred in 5 mL of di­chloro­ethane for 15 min. Isoamyl nitrite (0.32 g, 2.7 mmol) was added at 273–278 K. The reaction mixture was slowly brought to 298 K and allowed to stir for 1h. The reaction mixture was diluted with water and extracted with di­chloro­methane twice (2 × 50 mL) and concentrated to dryness to afford 1 g of crude product. The crude product was purified by flash chroma­tography using 2% methanol:di­chloro­methane eluent system to afford the title compound (0.35 g, 33%). ^1^H NMR (400 MHz, dimethyl sulfoxide): *δ* 9.70 (*s*, 1H), 7.47–7.48 (*d*, *J* = 2.8 Hz, 1H), 7.36 (*d*, *J* = 2.8 Hz, 1H), 4.41–4.42 (*m*, 1H), 4.08–4.11 (*m*, 2H), 2.97–3.04 (*m*, 4H), 2.51–2.58 (*m*, 2H), 1.67–1.98 (*m*, 11H), 1.42-1.46 (*m*, 2H), 1.24–1.28 (*m*, 1H), LC/MS (ESI) *m*/*e* 415.2 [*M* + H]^+^ calculated for C_20_H_27_N_6_O_4_. The crude product was dissolved in iso­octane-chloro­form (1:1 mixture) and kept at low temperature. After a week, single crystals were obtained.

## 7. Refinement

Crystal data, data collection and structure refinement details are summarized in Table 3 ▸. All of the hydrogen atoms, except H2 (attached to N2), which was located from a difference-Fourier map, were placed at their geometrically calculated positions and refined using a riding model with *U_i_*_so_(H) = 1.2*U*_eq_(C) or 1.5*U*_eq_(C-meth­yl).

## Supplementary Material

Crystal structure: contains datablock(s) I. DOI: 10.1107/S2056989025001793/tx2094sup1.cif

Structure factors: contains datablock(s) I. DOI: 10.1107/S2056989025001793/tx2094Isup5.hkl

CCDC reference: 2426771

Additional supporting information:  crystallographic information; 3D view; checkCIF report

## Figures and Tables

**Figure 5:**
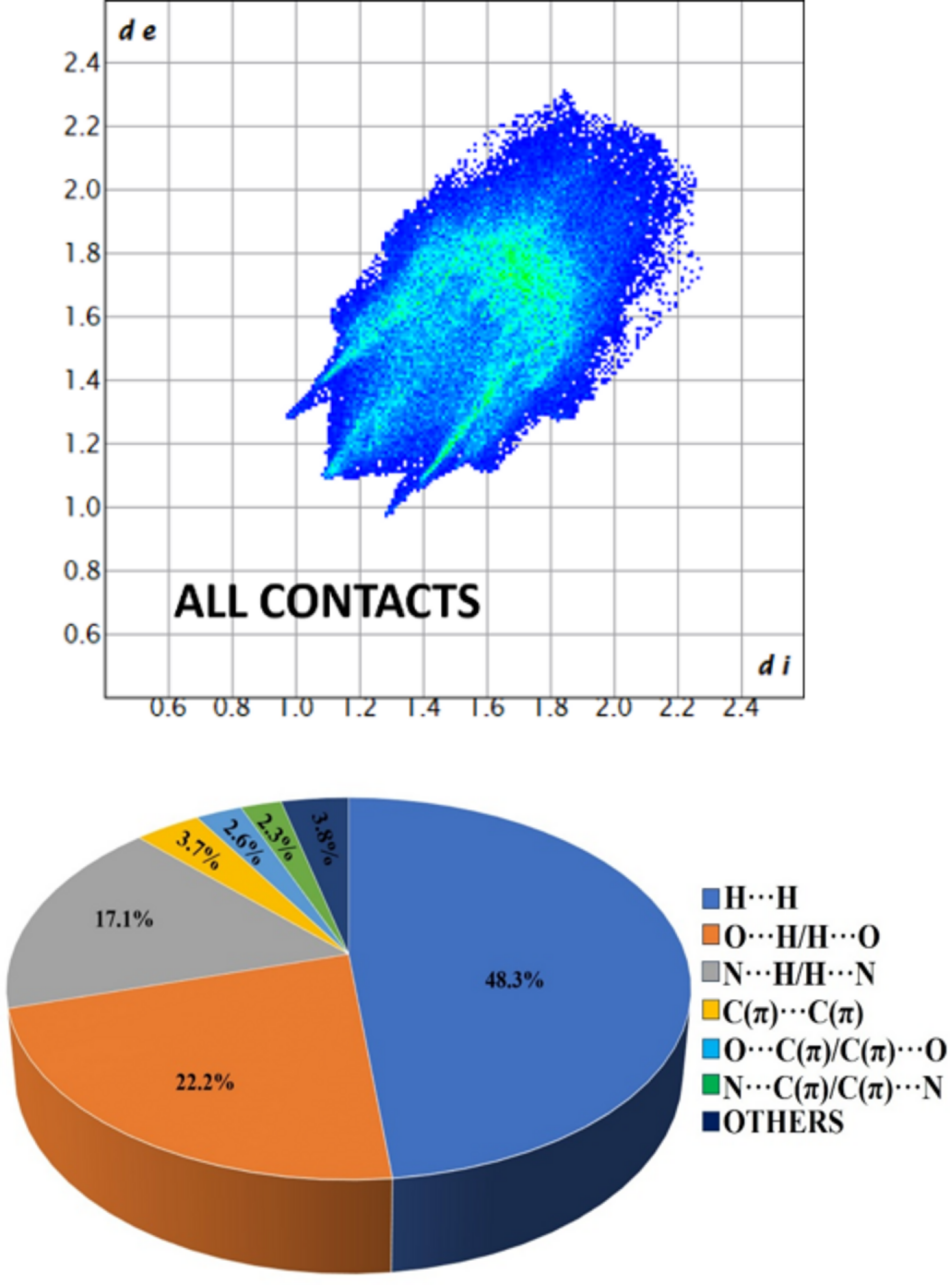
The fingerprint plot showing the overall contribution of all contacts and a diagram showing the percentage of individual contributions in the crystal packing.

**Table 1:** Torsion angles (°) of the but­oxy chain in **I**, **IA**, **IC** (see *Database survey*)

Torsion angle	**I**	**IA**	**IC**
τ_1_	177.7 (2)	−7.9 (2)	4.1 (2)
τ_2_	−179.4 (2)	174.6 (1)	175.8 (1)
τ_3_	−74.1 (2)	174.8 (1)	−174.7 (1)
τ_4_	−171.1 (2)	70.9 (1)	178.5 (1)
τ_5_	176.1 (2)	179.6 (1)	−64.6 (2)
τ_6_	168.9 (2)	−111.7 (1)	−94.9 (2)

**Table 2:** Inter­molecular inter­actions (Å, °) present in **I**

Motif	*D*—*X*⋯*Y*	*D*—*X*	*X*⋯*Y*	*D*⋯*Y*	∠*D*—*X*⋯*Y*
I^vi^	C8—H8*A*⋯O2—N1	0.99	2.71	3.467 (2)	133
	C3(π)⋯C1(π)	–	3.261 (3)	–	–
	N1⋯C5(π)	–	3.230 (3)	–	–
	C12—H12*B*⋯O3—C9	0.99	2.62	3.553 (3)	157
II^iii^	C10—H10*B*⋯O1—N1	0.99	2.74	3.526 (3)	136
	C3(π)⋯C1(π)	–	3.207 (3)	–	–
	N1⋯C5(π)	–	3.241 (3)	–	–
III^ii^	C17—H17⋯N15	1.00	2.73	3.705 (3)	165
	C13—H13*A*⋯N15	0.99	2.80	3.646 (3)	144
IV^vii^	C18—H18*A*⋯O1—N1	0.99	2.59	3.527 (3)	157
	C14(π)⋯O2	–	3.146 (3)	–	–
V^i^	C3—H3⋯O3—C9	0.95	2.75	3.681 (3)	168
	O4⋯C8—H8*B*	0.99	2.55	3.453 (3)	151
VI^v^	C8—H8*A*⋯O3—C9	0.99	2.57	3.409 (3)	142
VII^iv^	C7—H7*A*⋯O3—C9	0.99	2.35	3.170 (2)	140

Symmetry codes: (i) *x*, *y* + 1, *z*; (ii) *x* − 1, *y*, *z*; (iii) −*x*, −*y*, −*z* + 1; (iv) −*x*, −1 − *y*, 1 − *z*; (v) −*x* + 1, −*y* − 1, −*z* + 1; (vi) −*x* + 1, −*y*, −*z* + 1; (vii) −*x* + 1, −*y* + 1, −*z* + 1.

**Table 3:** Experimental details

Crystal data	
Chemical formula	C_20_H_26_N_6_O_4_
*M*_r_	414.47
Crystal system, space group	Triclinic, *P* 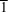
Temperature (K)	100
*a*, *b*, *c* (Å)	6.4597 (8), 9.2666 (11), 17.382 (2)
α, β, γ (°)	104.113 (5), 96.645 (5), 99.656 (5)
*V* (Å^3^)	981.3 (2)
*Z*	2
Radiation type	Mo *K*α
μ (mm^−1^)	0.10
Crystal size (mm)	0.24 × 0.13 × 0.05

Data collection	
Diffractometer	Bruker D8 Quest
Absorption correction	Multi-scan (*SADABS*; Krause *et al.*, 2015 ▸)
*T*_min_, *T*_max_	0.683, 0.746
No. of measured, independent and observed [*I* > 2σ(*I*)] reflections	16058, 5647, 4069
*R*_int_	0.043
(sin θ/λ)_max_ (Å^−1^)	0.705

Refinement	
*R*[*F*^2^ > 2σ(*F*^2^)], *wR*(*F*^2^), *S*	0.069, 0.141, 1.08
No. of reflections	5647
No. of parameters	275
H-atom treatment	H atoms treated by a mixture of independent and constrained refinement
Δρ_max_, Δρ_min_ (e Å^−3^)	0.36, −0.39

Computer programs: *APEX2* and *SAINT* (Bruker, 2016 ▸), *SHELXT2018/2* (Sheldrick, 2015*a* ▸), *SHELXL2018/3* (Sheldrick, 2015*b* ▸) and *OLEX2* (Dolomanov *et al.*, 2009 ▸).

## supplementary crystallographic information

### 6-[4-(1-Cyclohexyl-1H-tetrazol-5-yl)butoxy]-8-nitro-3,4-dihydroquinolin-2(1H)-one Crystal data

**Table:**

C_20_H_26_N_6_O_4_	*Z* = 2
*M**_r_* = 414.47	*F*(000) = 440
Triclinic, *P*1	*D*_x_ = 1.403 Mg m^−^^3^
*a* = 6.4597 (8) Å	Mo *K*α radiation, λ = 0.71073 Å
*b* = 9.2666 (11) Å	Cell parameters from 4187 reflections
*c* = 17.382 (2) Å	θ = 2.5–30.0°
α = 104.113 (5)°	µ = 0.10 mm^−^^1^
β = 96.645 (5)°	*T* = 100 K
γ = 99.656 (5)°	Plate, yellow
*V* = 981.3 (2) Å^3^	0.24 × 0.13 × 0.05 mm

### 6-[4-(1-Cyclohexyl-1H-tetrazol-5-yl)butoxy]-8-nitro-3,4-dihydroquinolin-2(1H)-one Data collection

**Table:**

Bruker D8 Quest diffractometer	5647 independent reflections
Radiation source: microfocus sealed X-ray tube, Incoatec Iµs	4069 reflections with *I* > 2σ(*I*)
Mirror optics monochromator	*R*_int_ = 0.043
Detector resolution: 7.9 pixels mm^-1^	θ_max_ = 30.1°, θ_min_ = 2.3°
ω and φ scans	*h* = −9→9
Absorption correction: multi-scan (SADABS; Krause *et al.*, 2015)	*k* = −12→13
*T*_min_ = 0.683, *T*_max_ = 0.746	*l* = −24→24
16058 measured reflections	

### 6-[4-(1-Cyclohexyl-1H-tetrazol-5-yl)butoxy]-8-nitro-3,4-dihydroquinolin-2(1H)-one Refinement

**Table:**

Refinement on *F*^2^	Primary atom site location: structure-invariant direct methods
Least-squares matrix: full	Hydrogen site location: mixed
*R*[*F*^2^ > 2σ(*F*^2^)] = 0.069	H atoms treated by a mixture of independent and constrained refinement
*wR*(*F*^2^) = 0.141	*w* = 1/[σ^2^(*F*_o_^2^) + (0.036*P*)^2^ + 1.0039*P*] where *P* = (*F*_o_^2^ + 2*F*_c_^2^)/3
*S* = 1.08	(Δ/σ)_max_ < 0.001
5647 reflections	Δρ_max_ = 0.36 e Å^−^^3^
275 parameters	Δρ_min_ = −0.39 e Å^−^^3^
0 restraints	

### 6-[4-(1-Cyclohexyl-1H-tetrazol-5-yl)butoxy]-8-nitro-3,4-dihydroquinolin-2(1H)-one Special details

**Table:**

Geometry. All esds (except the esd in the dihedral angle between two l.s. planes) are estimated using the full covariance matrix. The cell esds are taken into account individually in the estimation of esds in distances, angles and torsion angles; correlations between esds in cell parameters are only used when they are defined by crystal symmetry. An approximate (isotropic) treatment of cell esds is used for estimating esds involving l.s. planes.

### 6-[4-(1-Cyclohexyl-1H-tetrazol-5-yl)butoxy]-8-nitro-3,4-dihydroquinolin-2(1H)-one Fractional atomic coordinates and isotropic or equivalent isotropic displacement parameters (Å^2^)

**Table:**

	*x*	*y*	*z*	*U*_iso_*/*U*_eq_	
O4	0.3538 (2)	0.31868 (16)	0.63879 (8)	0.0143 (3)	
O3	0.2010 (2)	−0.53087 (16)	0.43484 (9)	0.0182 (3)	
O1	0.1523 (3)	−0.15696 (17)	0.33108 (8)	0.0196 (3)	
O2	0.2294 (2)	0.08708 (17)	0.35239 (9)	0.0189 (3)	
N1	0.2034 (3)	−0.0266 (2)	0.37737 (10)	0.0127 (3)	
N2	0.1868 (3)	−0.28422 (19)	0.45172 (10)	0.0120 (3)	
N3	1.0704 (3)	0.8204 (2)	0.79866 (10)	0.0163 (4)	
N4	0.9390 (3)	1.01503 (19)	0.85030 (10)	0.0132 (3)	
N15	1.2241 (3)	0.9502 (2)	0.81932 (11)	0.0195 (4)	
N16	1.1471 (3)	1.0679 (2)	0.85096 (11)	0.0184 (4)	
C4	0.3116 (3)	0.1701 (2)	0.59483 (11)	0.0113 (4)	
C6	0.2611 (3)	−0.1011 (2)	0.58111 (11)	0.0107 (4)	
C3	0.2731 (3)	0.1415 (2)	0.51206 (11)	0.0115 (4)	
H3	0.272801	0.223263	0.488058	0.014*	
C1	0.2277 (3)	−0.1325 (2)	0.49707 (11)	0.0108 (4)	
C5	0.3044 (3)	0.0472 (2)	0.62909 (11)	0.0114 (4)	
H5	0.329520	0.066015	0.685933	0.014*	
C2	0.2347 (3)	−0.0072 (2)	0.46401 (11)	0.0109 (4)	
C14	0.8946 (3)	0.8632 (2)	0.81830 (11)	0.0132 (4)	
C9	0.2296 (3)	−0.4038 (2)	0.48070 (12)	0.0130 (4)	
C17	0.7998 (3)	1.1125 (2)	0.88721 (12)	0.0137 (4)	
H17	0.650785	1.068434	0.858439	0.016*	
C10	0.3854 (3)	0.3463 (2)	0.72468 (11)	0.0147 (4)	
H10A	0.507798	0.304216	0.742582	0.018*	
H10B	0.257221	0.295982	0.741387	0.018*	
C7	0.2393 (3)	−0.2331 (2)	0.61839 (12)	0.0129 (4)	
H7A	0.088266	−0.265566	0.622944	0.015*	
H7B	0.323372	−0.199896	0.673263	0.015*	
C13	0.6805 (3)	0.7660 (2)	0.81081 (13)	0.0178 (4)	
H13A	0.572194	0.814652	0.787945	0.021*	
H13B	0.653491	0.763306	0.865403	0.021*	
C11	0.4277 (3)	0.5164 (2)	0.76270 (12)	0.0150 (4)	
H11A	0.408954	0.534190	0.819755	0.018*	
H11B	0.319495	0.559413	0.735488	0.018*	
C8	0.3162 (3)	−0.3673 (2)	0.56840 (12)	0.0145 (4)	
H8A	0.473662	−0.343760	0.575942	0.017*	
H8B	0.272399	−0.457746	0.588016	0.017*	
C18	0.8604 (4)	1.2733 (2)	0.87864 (13)	0.0184 (4)	
H18A	0.849021	1.270559	0.820994	0.022*	
H18B	1.009626	1.318310	0.904730	0.022*	
C12	0.6491 (3)	0.6027 (2)	0.75913 (12)	0.0156 (4)	
H12A	0.759748	0.552795	0.779382	0.019*	
H12B	0.661840	0.602030	0.702839	0.019*	
C22	0.8051 (4)	1.1126 (2)	0.97530 (13)	0.0178 (4)	
H22A	0.762243	1.007088	0.978963	0.021*	
H22B	0.951825	1.153979	1.004952	0.021*	
C21	0.6549 (4)	1.2086 (3)	1.01333 (13)	0.0198 (5)	
H21A	0.663914	1.210730	1.070855	0.024*	
H21B	0.506652	1.162545	0.986406	0.024*	
C20	0.7128 (4)	1.3703 (3)	1.00564 (13)	0.0204 (5)	
H20A	0.609442	1.429620	1.028136	0.024*	
H20B	0.855917	1.419567	1.036988	0.024*	
C19	0.7118 (4)	1.3706 (3)	0.91805 (13)	0.0212 (5)	
H19A	0.756991	1.476331	0.915033	0.025*	
H19B	0.565143	1.331179	0.888035	0.025*	
H2	0.150 (3)	−0.304 (2)	0.3988 (13)	0.009 (5)*	

### 6-[4-(1-Cyclohexyl-1H-tetrazol-5-yl)butoxy]-8-nitro-3,4-dihydroquinolin-2(1H)-one Atomic displacement parameters (Å^2^)

**Table:**

	*U*^11^	*U*^22^	*U*^33^	*U*^12^	*U*^13^	*U*^23^
O4	0.0222 (8)	0.0082 (7)	0.0122 (7)	0.0025 (6)	0.0026 (6)	0.0022 (5)
O3	0.0195 (8)	0.0090 (7)	0.0240 (8)	0.0019 (6)	0.0062 (6)	0.0001 (6)
O1	0.0264 (9)	0.0162 (8)	0.0129 (7)	0.0035 (7)	0.0015 (6)	−0.0008 (6)
O2	0.0257 (9)	0.0179 (8)	0.0157 (7)	0.0047 (7)	0.0037 (6)	0.0092 (6)
N1	0.0091 (8)	0.0150 (8)	0.0137 (8)	0.0029 (7)	0.0016 (6)	0.0031 (6)
N2	0.0133 (8)	0.0093 (8)	0.0116 (8)	0.0009 (6)	0.0013 (6)	0.0009 (6)
N3	0.0156 (9)	0.0174 (9)	0.0159 (8)	0.0055 (7)	0.0039 (7)	0.0023 (7)
N4	0.0120 (8)	0.0123 (8)	0.0151 (8)	0.0027 (7)	0.0035 (6)	0.0024 (6)
N15	0.0168 (9)	0.0205 (10)	0.0203 (9)	0.0049 (8)	0.0061 (7)	0.0019 (7)
N16	0.0123 (9)	0.0189 (9)	0.0225 (9)	0.0002 (7)	0.0046 (7)	0.0039 (7)
C4	0.0090 (9)	0.0095 (9)	0.0160 (9)	0.0032 (7)	0.0033 (7)	0.0032 (7)
C6	0.0088 (9)	0.0110 (9)	0.0143 (9)	0.0035 (7)	0.0045 (7)	0.0049 (7)
C3	0.0095 (9)	0.0110 (9)	0.0156 (9)	0.0020 (7)	0.0033 (7)	0.0059 (7)
C1	0.0077 (9)	0.0100 (9)	0.0151 (9)	0.0015 (7)	0.0038 (7)	0.0033 (7)
C5	0.0108 (9)	0.0128 (9)	0.0106 (8)	0.0013 (8)	0.0025 (7)	0.0033 (7)
C2	0.0075 (9)	0.0139 (10)	0.0112 (9)	0.0014 (7)	0.0014 (7)	0.0035 (7)
C14	0.0168 (10)	0.0118 (9)	0.0100 (8)	0.0037 (8)	0.0013 (7)	0.0010 (7)
C9	0.0093 (9)	0.0093 (9)	0.0215 (10)	0.0020 (7)	0.0066 (8)	0.0046 (8)
C17	0.0120 (10)	0.0110 (9)	0.0172 (9)	0.0035 (8)	0.0031 (8)	0.0009 (7)
C10	0.0205 (11)	0.0117 (10)	0.0111 (9)	0.0021 (8)	0.0025 (8)	0.0026 (7)
C7	0.0165 (10)	0.0083 (9)	0.0141 (9)	0.0012 (8)	0.0030 (8)	0.0042 (7)
C13	0.0150 (11)	0.0139 (10)	0.0213 (10)	0.0015 (8)	0.0043 (8)	−0.0008 (8)
C11	0.0197 (11)	0.0116 (10)	0.0131 (9)	0.0034 (8)	0.0041 (8)	0.0014 (7)
C8	0.0157 (10)	0.0108 (9)	0.0186 (10)	0.0025 (8)	0.0040 (8)	0.0064 (8)
C18	0.0255 (12)	0.0153 (10)	0.0179 (10)	0.0070 (9)	0.0080 (9)	0.0066 (8)
C12	0.0185 (11)	0.0104 (9)	0.0174 (10)	0.0043 (8)	0.0041 (8)	0.0016 (8)
C22	0.0225 (11)	0.0137 (10)	0.0216 (10)	0.0065 (9)	0.0092 (9)	0.0083 (8)
C21	0.0213 (12)	0.0216 (11)	0.0209 (11)	0.0097 (9)	0.0086 (9)	0.0077 (9)
C20	0.0277 (12)	0.0163 (11)	0.0186 (10)	0.0118 (9)	0.0045 (9)	0.0021 (8)
C19	0.0309 (13)	0.0162 (11)	0.0203 (10)	0.0096 (10)	0.0065 (9)	0.0078 (9)

### 6-[4-(1-Cyclohexyl-1H-tetrazol-5-yl)butoxy]-8-nitro-3,4-dihydroquinolin-2(1H)-one Geometric parameters (Å, º)

**Table:**

O4—C4	1.366 (2)	C10—H10B	0.9900
O4—C10	1.436 (2)	C10—C11	1.517 (3)
O3—C9	1.221 (2)	C7—H7A	0.9900
O1—N1	1.243 (2)	C7—H7B	0.9900
O2—N1	1.228 (2)	C7—C8	1.526 (3)
N1—C2	1.458 (2)	C13—H13A	0.9900
N2—C1	1.398 (2)	C13—H13B	0.9900
N2—C9	1.379 (2)	C13—C12	1.526 (3)
N2—H2	0.89 (2)	C11—H11A	0.9900
N3—N15	1.367 (3)	C11—H11B	0.9900
N3—C14	1.319 (3)	C11—C12	1.533 (3)
N4—N16	1.349 (2)	C8—H8A	0.9900
N4—C14	1.348 (3)	C8—H8B	0.9900
N4—C17	1.470 (3)	C18—H18A	0.9900
N15—N16	1.301 (3)	C18—H18B	0.9900
C4—C3	1.383 (3)	C18—C19	1.531 (3)
C4—C5	1.404 (3)	C12—H12A	0.9900
C6—C1	1.401 (3)	C12—H12B	0.9900
C6—C5	1.386 (3)	C22—H22A	0.9900
C6—C7	1.511 (3)	C22—H22B	0.9900
C3—H3	0.9500	C22—C21	1.523 (3)
C3—C2	1.391 (3)	C21—H21A	0.9900
C1—C2	1.412 (3)	C21—H21B	0.9900
C5—H5	0.9500	C21—C20	1.525 (3)
C14—C13	1.491 (3)	C20—H20A	0.9900
C9—C8	1.496 (3)	C20—H20B	0.9900
C17—H17	1.0000	C20—C19	1.522 (3)
C17—C18	1.524 (3)	C19—H19A	0.9900
C17—C22	1.527 (3)	C19—H19B	0.9900
C10—H10A	0.9900		
			
C4—O4—C10	116.53 (15)	C14—C13—H13A	108.4
O1—N1—C2	119.39 (16)	C14—C13—H13B	108.4
O2—N1—O1	121.98 (17)	C14—C13—C12	115.69 (18)
O2—N1—C2	118.62 (17)	H13A—C13—H13B	107.4
C1—N2—H2	117.8 (14)	C12—C13—H13A	108.4
C9—N2—C1	124.94 (17)	C12—C13—H13B	108.4
C9—N2—H2	116.4 (14)	C10—C11—H11A	108.5
C14—N3—N15	105.82 (17)	C10—C11—H11B	108.5
N16—N4—C17	122.55 (17)	C10—C11—C12	114.99 (17)
C14—N4—N16	108.70 (17)	H11A—C11—H11B	107.5
C14—N4—C17	128.46 (18)	C12—C11—H11A	108.5
N16—N15—N3	110.93 (17)	C12—C11—H11B	108.5
N15—N16—N4	106.10 (17)	C9—C8—C7	112.55 (17)
O4—C4—C3	117.16 (17)	C9—C8—H8A	109.1
O4—C4—C5	123.72 (17)	C9—C8—H8B	109.1
C3—C4—C5	119.11 (18)	C7—C8—H8A	109.1
C1—C6—C7	118.46 (17)	C7—C8—H8B	109.1
C5—C6—C1	120.91 (18)	H8A—C8—H8B	107.8
C5—C6—C7	120.58 (17)	C17—C18—H18A	109.8
C4—C3—H3	120.1	C17—C18—H18B	109.8
C4—C3—C2	119.86 (18)	C17—C18—C19	109.59 (18)
C2—C3—H3	120.1	H18A—C18—H18B	108.2
N2—C1—C6	118.42 (17)	C19—C18—H18A	109.8
N2—C1—C2	124.44 (17)	C19—C18—H18B	109.8
C6—C1—C2	117.13 (18)	C13—C12—C11	108.81 (17)
C4—C5—H5	119.5	C13—C12—H12A	109.9
C6—C5—C4	120.92 (18)	C13—C12—H12B	109.9
C6—C5—H5	119.5	C11—C12—H12A	109.9
C3—C2—N1	116.24 (17)	C11—C12—H12B	109.9
C3—C2—C1	122.02 (17)	H12A—C12—H12B	108.3
C1—C2—N1	121.74 (17)	C17—C22—H22A	109.6
N3—C14—N4	108.44 (18)	C17—C22—H22B	109.6
N3—C14—C13	128.13 (19)	H22A—C22—H22B	108.1
N4—C14—C13	123.40 (18)	C21—C22—C17	110.43 (18)
O3—C9—N2	119.85 (19)	C21—C22—H22A	109.6
O3—C9—C8	123.46 (19)	C21—C22—H22B	109.6
N2—C9—C8	116.67 (17)	C22—C21—H21A	109.5
N4—C17—H17	108.0	C22—C21—H21B	109.5
N4—C17—C18	111.43 (17)	C22—C21—C20	110.50 (18)
N4—C17—C22	109.98 (17)	H21A—C21—H21B	108.1
C18—C17—H17	108.0	C20—C21—H21A	109.5
C18—C17—C22	111.33 (17)	C20—C21—H21B	109.5
C22—C17—H17	108.0	C21—C20—H20A	109.5
O4—C10—H10A	109.9	C21—C20—H20B	109.5
O4—C10—H10B	109.9	H20A—C20—H20B	108.0
O4—C10—C11	108.84 (16)	C19—C20—C21	110.93 (18)
H10A—C10—H10B	108.3	C19—C20—H20A	109.5
C11—C10—H10A	109.9	C19—C20—H20B	109.5
C11—C10—H10B	109.9	C18—C19—H19A	109.3
C6—C7—H7A	109.3	C18—C19—H19B	109.3
C6—C7—H7B	109.3	C20—C19—C18	111.82 (18)
C6—C7—C8	111.54 (16)	C20—C19—H19A	109.3
H7A—C7—H7B	108.0	C20—C19—H19B	109.3
C8—C7—H7A	109.3	H19A—C19—H19B	107.9
C8—C7—H7B	109.3		
			
O4—C4—C3—C2	178.34 (18)	C1—N2—C9—C8	3.3 (3)
O4—C4—C5—C6	−179.89 (18)	C1—C6—C5—C4	1.3 (3)
O4—C10—C11—C12	−74.1 (2)	C1—C6—C7—C8	34.9 (2)
O3—C9—C8—C7	−151.93 (19)	C5—C4—C3—C2	−2.1 (3)
O1—N1—C2—C3	−174.48 (18)	C5—C6—C1—N2	179.37 (18)
O1—N1—C2—C1	6.0 (3)	C5—C6—C1—C2	−1.6 (3)
O2—N1—C2—C3	5.7 (3)	C5—C6—C7—C8	−147.74 (19)
O2—N1—C2—C1	−173.81 (18)	C14—N3—N15—N16	0.5 (2)
N2—C1—C2—N1	−1.5 (3)	C14—N4—N16—N15	0.6 (2)
N2—C1—C2—C3	179.02 (19)	C14—N4—C17—C18	−152.15 (19)
N2—C9—C8—C7	29.8 (2)	C14—N4—C17—C22	83.9 (2)
N3—N15—N16—N4	−0.7 (2)	C14—C13—C12—C11	176.09 (17)
N3—C14—C13—C12	−13.5 (3)	C9—N2—C1—C6	−17.8 (3)
N4—C14—C13—C12	168.87 (18)	C9—N2—C1—C2	163.28 (19)
N4—C17—C18—C19	−179.86 (17)	C17—N4—N16—N15	175.00 (17)
N4—C17—C22—C21	−177.90 (18)	C17—N4—C14—N3	−174.26 (18)
N15—N3—C14—N4	−0.1 (2)	C17—N4—C14—C13	3.8 (3)
N15—N3—C14—C13	−178.08 (19)	C17—C18—C19—C20	55.8 (3)
N16—N4—C14—N3	−0.3 (2)	C17—C22—C21—C20	−57.1 (2)
N16—N4—C14—C13	177.78 (18)	C10—O4—C4—C3	177.68 (17)
N16—N4—C17—C18	34.6 (3)	C10—O4—C4—C5	−1.9 (3)
N16—N4—C17—C22	−89.3 (2)	C10—C11—C12—C13	−171.11 (17)
C4—O4—C10—C11	−179.44 (17)	C7—C6—C1—N2	−3.3 (3)
C4—C3—C2—N1	−177.77 (17)	C7—C6—C1—C2	175.74 (17)
C4—C3—C2—C1	1.8 (3)	C7—C6—C5—C4	−175.97 (18)
C6—C1—C2—N1	179.61 (18)	C18—C17—C22—C21	58.1 (2)
C6—C1—C2—C3	0.1 (3)	C22—C17—C18—C19	−56.7 (2)
C6—C7—C8—C9	−47.1 (2)	C22—C21—C20—C19	56.3 (3)
C3—C4—C5—C6	0.5 (3)	C21—C20—C19—C18	−56.1 (3)
C1—N2—C9—O3	−175.10 (18)		
